# Supplementary figures and images for: Reducing the Risk of Benthic Algae Outbreaks by Regulating the Flow Velocity in a Simulated South–North Water Diversion Open Channel
Source: Int J Environ Res Public Health. 2023 Feb 17;20(4):3564. doi: 10.3390/ijerph20043564 (PMC9966353; doi:10.3390/ijerph20043564)

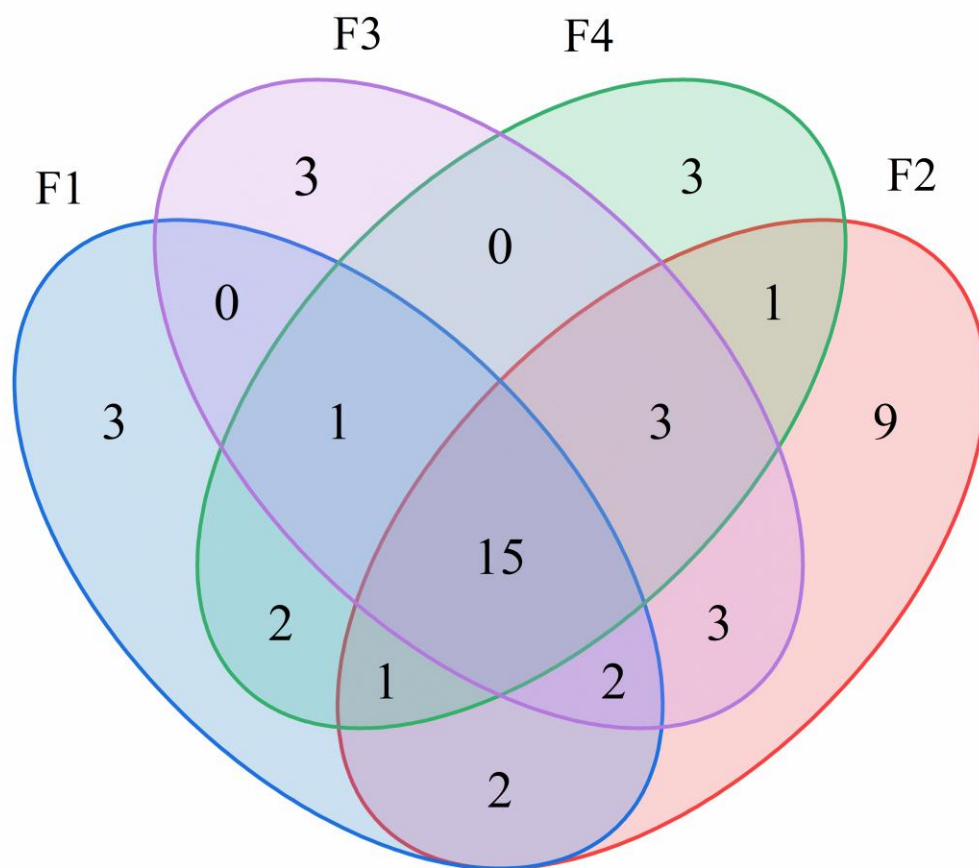

**Figure S1.** Differences in the number of species between each channel section.

Supplement: Supplementary file 1 [file ijerph-20-03564-s001.zip › ijerph-2145776-supplementary.pdf]
